# Arylsulfatase A Remodeling during Human Sperm In Vitro Capacitation Using Field Emission Scanning Electron Microscopy (FE-SEM)

**DOI:** 10.3390/cells10020222

**Published:** 2021-01-23

**Authors:** María José Gómez-Torres, Natalia Huerta-Retamal, Laura Robles-Gómez, Paula Sáez-Espinosa, Jon Aizpurua, Manuel Avilés, Alejandro Romero

**Affiliations:** 1 Departamento de Biotecnología, Universidad de Alicante, 03690 Alicante, Spain; natalia.huerta@ua.es (N.H.-R.); laura.robles@ua.es (L.R.-G.); paula.saez@ua.es (P.S.-E.); arr@ua.es (A.R.); 2 Cátedra Human Fertility, Universidad de Alicante, 03080 Alicante, Spain; j.aizpurua@ivf-spain.com; 3 IVF Spain, Medicina Reproductiva, 03540 Alicante, Spain; 4 Departamento de Biología Celular e Histología, Universidad de Murcia, Instituto Murciano de Investigación Sociosanitaria (IMIB), 30003 Murcia, Spain; maviles@um.es

**Keywords:** spermatozoa, capacitation, ARSA, FE-SEM, gold-labeling

## Abstract

Capacitation drives sperm biophysical and biochemical changes for sperm-oocyte interactions. It is a well-known fact that the molecular complex arylsulfatase A (ARSA), hyaluronidase sperm adhesion molecule 1 (SPAM1), and heat shock protein 2 (HSPA2) plays a significant role in sperm–zona pellucida (ZP) binding. However, the time-dependent capacitation effects on the sperm surface ARSA presence and specific topographic distributions remain to be elucidated. Here, we quantified the ARSA density and specific membrane domain locations before (US) and after in vitro capacitation (one and four hours; CS1–CS4) in human sperm using high-resolution field emission scanning electron microscopy (FE-SEM) and immunogold labeling. Our results showed a significant and progressive capacitation-mediated increase of labeled spermatozoa from the US (37%) to CS4 (100%) physiological conditions. In addition, surface mapping revealed a close relationship between the ARSA residues and their acrosomal repositioning. Compared with the ARSA surface heterogeneous distribution found in US, the CS1–4 conditions exhibited clustering on the peri-acrosomal region, showing that time-dependent capacitation also induced a ARSA residue dramatic translocation on sperm surfaces. Our findings provide novel insights into the molecular remodeling events preceding sperm-oocyte interactions.

## 1. Introduction

Prior to fertilization success, capacitation provides the spermatozoa with the ability to interact and fuse with the oocyte plasma membrane [1], which involves the reorganization of molecular receptors needed for a sperm-oocyte interaction [2,3] 

The protein complex constituted by arylsulfatase A (ARSA), heat shock protein A2 (HSPA2), and hyaluronidase sperm adhesion molecule 1 (SPAM1) is a molecular receptor complex involved in sperm-oocyte recognition and is crucial for male fertility [2]. In this context, using immunofluorescence techniques, the ARSA presence in human capacitated spermatozoa and its colocalization with the proteins of the complex (HSPA2/SPAM1) in the peri-acrosomal region is proposed as the mediator of sperm–zona pellucida (ZP) binding [3]. Moreover, a significant increase of ARSA in capacitated spermatozoa, mainly located in the acrosome domain, has also been recorded by immunofluorescence [4]. Therefore, ARSA quantitative detection and distribution during in vitro sperm capacitation could be used to get a better insight into molecular changes during the fertilization process and improve artificial reproductive technologies. To achieve this, immunogold labeling for field emission scanning electron microscopy (FE-SEM) provides a high-resolution tool for the detection and quantification of surface molecules in plasma membranes [5,6], allowing a protein characterization with even higher-detail resolution than previous immunofluorescence studies [3,4]. For that, the aim of this study was to analyze the density and topographic distribution of ARSA by FE-SEM during time-dependent (one- and four-hour) in vitro capacitation in human spermatozoa.

## 2. Materials and Methods

### 2.1. Seminal Sample Analysis

Semen samples were obtained by masturbation from five donors after three-to-four days of sexual abstinence. A basic semen analysis was performed prior to one hour, and all the samples included were classified as normozoospermic according to the World Health Organization [7]. The samples were then divided into three aliquots in order to be studied before (uncapacitated sperm; US) and after one (one-hour-capacitated sperm; CS1) and four hours (four-hour capacitated sperm; CS4) of in vitro capacitation. This study was approved by the Bioethics Committee of the University of Alicante (Alicante, Spain) in accordance with the Declaration of Helsinki principles, and informed consent was obtained from each donor.

### 2.2. In Vitro Capacitation

The seminal plasma was removed by centrifugation for ten minutes at 250× *g*; formerly, a wash in a human tubal fluid medium (HTF, Origio^®^, Måløv, Denmark) for five minutes at 250× *g* was made. The cells were then capacitated using HTF medium supplemented with 5 mg/mL of bovine serum albumin (BSA; Sigma-Aldrich^®^, Saint Louis, MO, USA) at 37 °C and 5% (*v*/*v*) CO_2_. The motile sperm fraction was recovered by the swim-up technique after one and four hours [7] and subsequently washed in phosphate-buffered saline (PBS; Biowest^®^, Nuaillé, France). To corroborate the sperm capacitation, the presence of phosphorylated tyrosine in the sperm flagellum was evaluated following the methodology described in previous studies [8].

### 2.3. Fixation

For FE-SEM immunogold labeling, all experimental samples were previously fixed in 2.5% (*w*/*v*) glutaraldehyde containing 2% (*w*/*v*) paraformaldehyde (Electron Microscopy Sciences, Hatfield, PA, USA) for one hour at 4 °C. Finally, the fixative solution was removed, and cells were resuspended in PBS to reach a final concentration of 1 mill/mL and stored at 4 °C until use.

### 2.4. FE-SEM Immunogold Labeling

To evaluate the ARSA location by FE-SEM, we followed the methods previously described by our group [4,6]. A total of 5 µl of fixed samples were placed on coverslips and, once dried, washed in distilled water (2 × 2 min) and rehydrated in PBS (3 × 5 min). After washing, cells were incubated with the primary anti-ARSA antibody (Sigma-Aldrich^®^, Sant Louis, MO, USA) at a final optimal concentration of 1:10 in blocking solution for two hours at 37 °C in a humid chamber. Due to the novelty of this technique, the primary antibody concentration was selected as optimal after the implementation of a concentration curve based on the commonly used concentration for immunofluorescence studies [3,9].

After three washes in PBS (3 × 5 min), the coverslips were incubated with gold-conjugated protein A (10 nm) (British Biocell International, Cardiff, United Kingdom) in a blocking solution (2% BSA in PBS) at a final optimal concentration of 1:50 for one hour at room temperature. Since particles >20 nm in size mask sample details for scanning microscopy [10], the diameter of the gold nanoparticles used here allowed the observation of large surfaces with good sensitivity and precision (see [6] for further details).

Coverslips were then washed three times in PBS (3 × 5 min) and post-fixed in 1% (*w*/*v*) glutaraldehyde for 30 min. Finally, the cells were washed three times in PBS and twice in distilled H_2_O before being critical point-dried [11] and mounted in stubs with carbon tape, which acted as a conductive element. Negative control experiments were performed omitting the anti-ARSA antibody. The absence of gold particles verified the specificity of the immunolabeling.

### 2.5. FE-SEM Imaging and Gold Quantification

Samples were coated by an ultra-thin layer of carbon (SCD 004 Sputter Coater; Bal-Tec AG, Balzers, Liechtenstein) and examined using a FE-SEM Zeiss Merlin VP Compact. The FE-SEM was set to a voltage of 2 kV and EHT (extra-high tension) mode [6,10]. A total of 250 individual spermatozoa head digital micrographs (1024 × 768 pixels) were randomly obtained at a standardized 18,000× magnification for each physiological condition (US, CS1, and CS4) using the InlensDuo signal combining both the secondary and backscattered electron emission modes [6]. FE-SEM micrographs were then edited using Adobe Photoshop^®^ CS5 to obtain oriented spermatozoa heads in a same Cartesian position, adding a digital grid layer (17 × 25 square units) overlaid over each sperm head. SigmaScan^®^ Pro (SPSS^TM^, Chicago, IL, USA) digital imaging software was used to count the gold nanoparticles and recorded their topographic spatial distribution to generate wafer maps.

### 2.6. Statistical Analysis

The Shapiro-Wilk test showed that the gold nanoparticle counts were not normally distributed (W = 0.8; *p* < 0.001) for each sperm physiological condition (US, CS1, and CS4). The nonparametric Kruskal-Wallis test was then computed to determine the significant labeling differences between the uncapacitated (US) and time-dependent in vitro capacitate (CS1 and CS4) spermatozoa. Descriptive (mean ± standard deviation) and statistical procedures were conducted using Statistica^®^ 10.0 (StatSoft^®^, Germany, Europe). The significance level was set at α = 0.05. 

## 3. Results and Discussion

Tyrosine phosphorylation was analyzed before and after different incubation times to support the success of sperm capacitation. Our results showed a higher percentage of spermatozoa with flagellar phosphorylated tyrosine in the CS4 subpopulation (32.4%) compared to those before and after one-hour capacitation (7.7% and 14.3%, respectively; *p* < 0.01). This time-dependent increase is consistent with previous studies that confirmed the usefulness of tyrosine phosphorylation as a capacitation biomarker [12,13].

**Figure 1:**
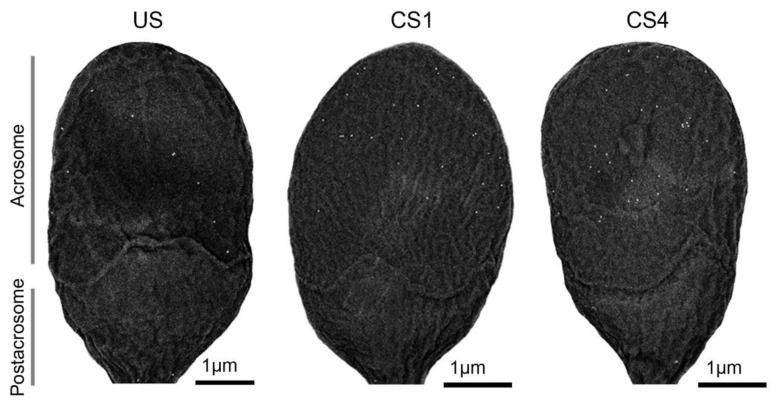
Human sperm head micrographs by field emission scanning electron microscopy (FE-SEM) showing the gold nanoparticle density and distribution variations among uncapacitated sperm (US) and after one (CS1) to four hours (CS4) of capacitation. Note that the gold nanoparticles are mainly located in the acrosomal region. A standardized 18,000× magnification was common to all sperm head micrographs.

After evaluating the FE-SEM micrographs, we found that the human sperm gold nanoparticle presence, mainly located across the acrosomal region, and density were time-dependent during the in vitro capacitation (Figure 1). The lower frequency of labeled uncapacitated spermatozoa (US: 37.2%; *n* = 93/250) contrasted with <3% of the nonlabeled spermatozoa during one (CS1: 97.6%; *n* = 244/250) to four hours (CS4: 100%; *n* = 250/250) of capacitation time. Accordingly, the ARSA, as a ZP putative receptor, underwent a capacitation-associated dramatic translocation to the sperm surface [3]. Early reports using flow cytometry and immunofluorescence techniques with prior antigenic retrieval [3,4,14] found frequencies (<20%) in the presence of ARSA before capacitation. Instead, our results recorded using higher-detail resolution FE-SEM techniques [6] allowed to characterize a higher frequency (>35%) of mark, ed spermatozoa. 

**Figure 2:**
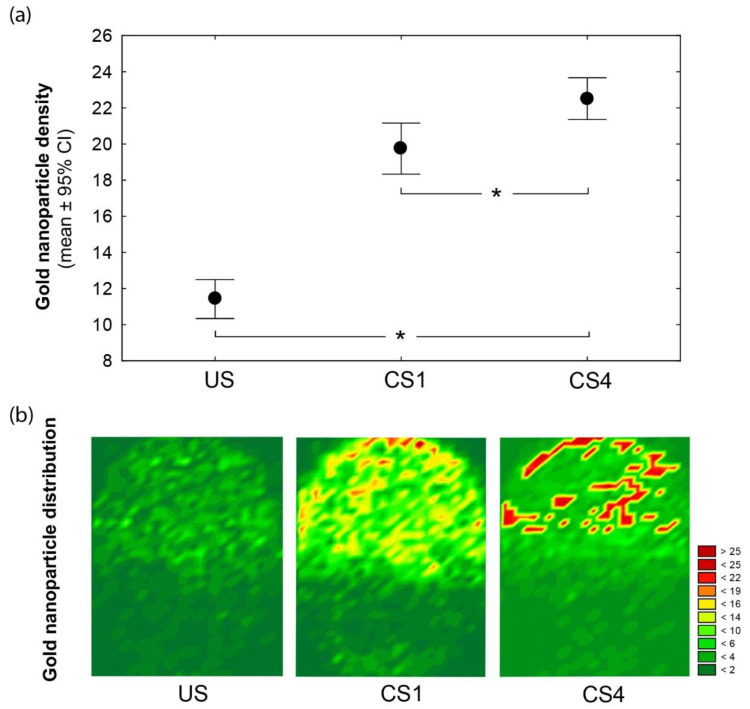
(**a**) The gold nanoparticle density and distribution differ among head spermatozoa according to the time-dependent capacitation conditions: uncapacitated (US), one- (CS1), and four-hour-capacitated (CS4) spermatozoa. Mean (dot) and whiskers denote ±95% confidence intervals (CI). Significant differences between the physiological conditions (Kruskal-Wallis at *p* < 0.05) are indicated (*). (**b**) Wafer maps illustrating the arylsulfatase A (ARSA) redistribution before (US) and after (CS1−4) spermatozoa capacitation. Topographic views denote the superposition of nanoparticle counts and their Cartesian spatial distributions from the labeled spermatozoa of each condition. The color scale indicates the nanoparticle count.

Moreover, a novel combined approach, by using FE-SEM and immunogold labeling, enabled a high-detail resolution and specific ARSA location on the spermatozoa heads. In turn, when only labeled spermatozoa are considered, we found that noncapacitated (US) spermatozoa exhibited significant lower ARSA density (US: 11.4 ± 5.2) than those recorded for one (CS1: 19.7 ± 11.2; *χ*^2^ = 55.6, *p* < 0.001) and four hours (CS4: 22.5 ± 9.2; *χ*^2^ = 121.3, *p* < 0.001) of capacitation. Therefore, a significant increase in the labeling densities from uncapacitated (US) to time-dependent in vitro capacitated (CS1-4: *χ*^2^ = 20.85, *p* < 0.001) spermatozoa were detected (Figure 2a). 

After in vitro capacitation, we also found a remarkable relocation of ARSA residues (Figure 2b). Surface mapping revealed that ARSA for the US spermatozoa were heterogeneously distributed and covered most of the acrosomal membrane surfaces. Instead, independently of the incubation time (one and four hours), spermatozoa showed the dynamics of ARSA, which was mainly clustered in the most apical (peri-acrosomal) region of the acrosome (see Figure 2b; CS1 and CS4). Overall, our findings support the relocation of the protein complex ARSA/HSPA2/SPAM1 to a preferential peri-acrosomal location [2,3,4]. Further, previous proteomic analyses have shown the presence of many candidate ZP-binding proteins in lipid rafts in this localization [15,16]. 

## 4. Conclusions

In summary, the FE-SEM-immunogold labeling techniques allowed us to properly quantify the ARSA changes in the density and location, which were time-dependent on the capacitation. This combined approach also illustrated the relevance of FE-SEM techniques to identify and characterize ZP-binding protein complexes in preparation for sperm-oocyte recognition.

## Data Availability

The data presented in this study are available in the article.

## Abbreviations

ARSA	Arylsulfatase A
BSA	Bovine serum albumin
CS1	One-hour capacitated sperm
CS4	Four-hour capacitated sperm
FE-SEM	Field emission scanning electron microscopy
HSPA2	Heat shock protein A2
HTF	Human tubal fluid
PBS	Phosphate-buffered saline
SPAM1	Sperm adhesion molecule 1
US	Uncapacitated sperm
ZP	Zona pellucida
